# Gesture Recognition Based on a Convolutional Neural Network–Bidirectional Long Short-Term Memory Network for a Wearable Wrist Sensor with Multi-Walled Carbon Nanotube/Cotton Fabric Material

**DOI:** 10.3390/mi15020185

**Published:** 2024-01-26

**Authors:** Yang Song, Mengru Liu, Feilu Wang, Jinggen Zhu, Anyang Hu, Niuping Sun

**Affiliations:** 1 School of Electronic and Information Engineering, Anhui Jianzhu University, Hefei 230601, China; esunny@ahjzu.edu.cn (Y.S.); lmr@stu.ahjzu.edu.cn (M.L.); zjg@stu.ahjzu.edu.cn (J.Z.); hay0610@stu.ahjzu.edu.cn (A.H.); snp@stu.ahjzu.edu.cn (N.S.); 2 Key Laboratory of Building Information Acquisition and Measurement Control Technology, Anhui Jianzhu University, Hefei 230601, China

**Keywords:** multi-walled carbon nanotube (MWCNT), pressure sensor unit, wrist sensor, gesture recognition, convolutional neural network (CNN), bidirectional long short-term memory (BiLSTM) neural network

## Abstract

Flexible pressure sensors play a crucial role in detecting human motion and facilitating human–computer interaction. In this paper, a type of flexible pressure sensor unit with high sensitivity (2.242 kPa^−1^), fast response time (80 ms), and remarkable stability (1000 cycles) is proposed and fabricated by the multi-walled carbon nanotube (MWCNT)/cotton fabric (CF) material based on a dip-coating method. Six flexible pressure sensor units are integrated into a flexible wristband and made into a wearable and portable wrist sensor with favorable stability. Then, seven wrist gestures (Gesture Group #1), five letter gestures (Gesture Group #2), and eight sign language gestures (Gesture Group #3) are performed by wearing the wrist sensor, and the corresponding time sequence signals of the three gesture groups (#1, #2, and #3) from the wrist sensor are collected, respectively. To efficiently recognize different gestures from the three groups detected by the wrist sensor, a fusion network model combined with a convolutional neural network (CNN) and the bidirectional long short-term memory (BiLSTM) neural network, named CNN-BiLSTM, which has strong robustness and generalization ability, is constructed. The three types of Gesture Groups were recognized based on the CNN-BiLSTM model with accuracies of 99.40%, 95.00%, and 98.44%. Twenty gestures (merged by Group #1, #2, and #3) were recognized with an accuracy of 96.88% to validate the applicability of the wrist sensor based on this model for gesture recognition. The experimental results denote that the CNN-BiLSTM model has very efficient performance in recognizing different gestures collected from the flexible wrist sensor.

## 1. Introduction

Human sensing interfaces have become a fundamental tool for capturing human physiological parameters. In recent years, the development of flexible wearable e-skin has spurred research on softness [1], comfort [2], breathability [3], biocompatibility [4,5], and stability [6]. To date, flexible pressure/strain sensors have been designed based on a variety of sensing mechanisms, including piezoelectric [7,8], capacitive [9,10], piezoresistive [11,12], and self-powered [13,14] sensing. Flexible pressure sensors based on the piezoresistive mechanism have high sensitivity and fast response time, making them widely used in detecting dynamically changing pressure signals [15]. The principle of piezoresistive sensors is to reflect variation in pressure by measuring changes in resistance between layers. The fabrics/textiles have attracted attention and have been used in a wide variety of applications, as they are flexible, environmentally friendly, mechanically stable, porous, low cost, and biocompatible. Piezoresistive fabric sensors have been widely studied [16,17,18,19]. Sensitive materials can be uniformly coated on the flexible substrate material by the dip-coating method to form the sensitive layer of the sensor [20,21,22,23,24,25]. The dip-coating method is a simple and easy-to-implement preparation method that does not require complex equipment or processing procedures. The conductive composites prepared by the dip-coating method have the advantages of simplicity, conductivity, flexibility, light weight, stability, and versatility.

A series of wearable sensors made from textiles and fabric have been proposed with great performance and have a wide range of applications in the areas of smart skin, human–computer interaction, and health detection. Zhou et al. [26] prepared stress and strain sensors by 3D printing composite conductive ink to produce high-performance smart gloves, which combined with deep learning to realize ultra-high-precision dynamic gesture recognition and control operations. Seesaard et al. [27] presented a flexible fabric-based piezoresistive force sensor with a layered structure prepared based on a key nanocomposite between Ti_3_AlC_2_ and PEDOT: PSS, whose excellent properties make it suitable for detecting a wide range of physiological data. Uno et al. [28] presented sensor yarns of carbon-coated multifibers covered with metal core yarns to detect pressure and strain. They can be integrated into fabrics and knitwear to detect external forces and biological motions. Zhou et al. [29] used a one-step screen printing technique to transfer graphene nanosheets (GNSs)/multi-walled carbon nanotube (MWCNT) hybrid inks onto a stretchable fabric tape to prepare a high-performance sensor and integrated a sensing glove based on the sensor, which was combined with an LSTM model to achieve gesture recognition with an accuracy of 95%. Zeng et al. [30] prepared a pure carbon-based wearable electronic textile (e-textile) by depositing the mixed ink of graphene and carbon nanotubes through the screen-printing process; five sensors were integrated into the fabric glove combined with machine learning, which can recognize eight different gestures with an average accuracy of 96.58%. Song et al. [31] proposed a graphene-coated silk-spandex (GCSS) fabric strain sensor prepared by reducing graphene oxide and knitting ten strain sensors on a smart glove to recognize five sign language gestures with an accuracy of 96.07%. Peng et al. [32] proposed a physiologically flexible band of graphene aerogel (GA) pressure sensors and attached it to the back of the hand. By sensing the tendon deformation on the back of the hand, a machine learning method was used to accurately identify 12 typical grasping gestures with an accuracy of 84.7%. Accurate detection and recognition of gesture movements can be achieved using wearable devices.

In our work, a type of flexible piezoresistive pressure sensor unit is proposed based on the multi-walled carbon nanotube (MWCNT)/cotton fabric (CF) composite (MWCNT/CF). A wearable and portable wrist sensor is fabricated by integrating six MWCNT/CF sensor units on a flexible fabric wristband. Twenty different gestures are executed by wearing the wrist sensor, and time sequence data corresponding to the gestures are collected. Then, a fusion model of the convolutional neural network (CNN) and the bidirectional long short-term memory (BiLSTM) neural network, named CNN-BiLSTM, is constructed to distinguish different gestures based on the time sequence signals, and the average recognition accuracy of 20 gestures is 96.88%. Gesture recognition results from references [29,30,31,32] and our work is based on different materials and different models that are listed in Table 1, which shows that the method proposed in this work has better performance in gesture recognition.

## 2. Fabrication Procedure

### 2.1. Structure of the MWCNT/CF Sensor Unit

As the multi-walled carbon nanotube (MWCNT) with excellent piezoresistive effects and high sensitivity and the cotton fabric (CF) have the advantages of being light in weight and good flexibility, and this work proposes an eco-friendly, cost-effective, and efficient method to fabricate piezoresistive sensor units using the MWCNT/CF composite as the sensitive element of the piezoresistive layer.

**Figure 1:**
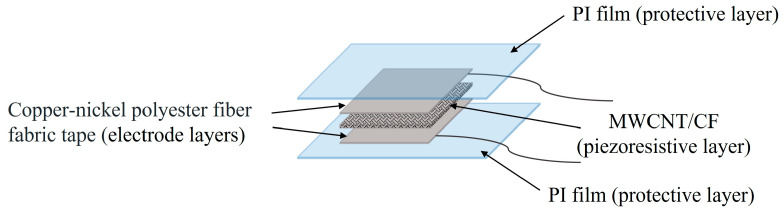
Structure of the MWCNT/CF sensor unit.

The structure of the MWCNT/CF sensor unit consists of a piezoresistive layer, two protective layers, and two electrode layers, as illustrated in Figure 1. The protective layers are primarily composed of polyimide (PI) film to protect the sensor unit from being worn and destroyed. The piezoresistive layer, which is the main component of the sensor unit, is predominantly composed of the MWCNT/CF composite. The upper and lower electrodes are made of the copper–nickel polyester fiber fabric tape.

### 2.2. Fabrication of the MWCNT/CF Sensor Unit

#### 2.2.1. Preparation of the MWCNT/CF Composite

**Figure 2:**
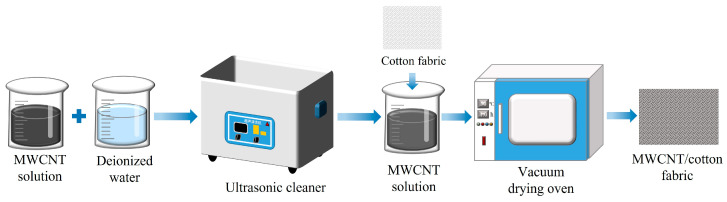
Fabrication of conductive cotton fabric based on the MWCNT.

**Figure 3:**
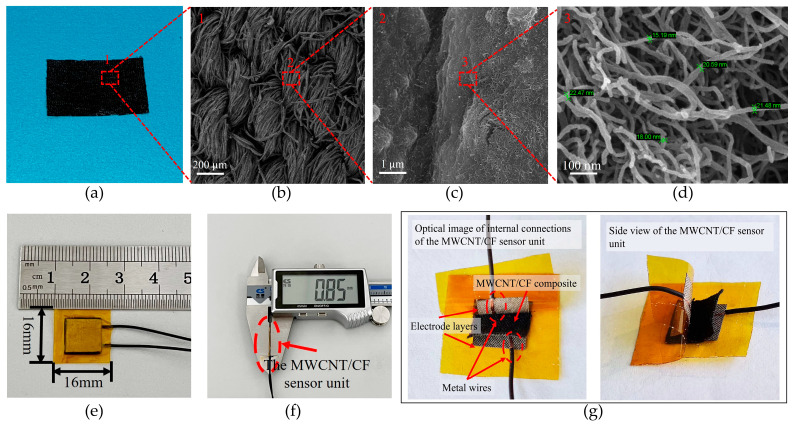
The SEM of the MWCNT/CF composite and the prototype of the MWCNT/CF sensor unit. (**a**) The MWCNT/CF composite (the red lines point to the enlarged area); (**b**) area 1 with SEM micrograph of the MWCNT/CF composite; (**c**) area 2 with magnified SEM micrograph of the MWCNT/CF composite; (**d**) area 3 with SEM micrograph of MWCNTs attached to cotton fibers; (**e**) the size of the sensor unit; (**f**) the thickness of the sensor unit; (**g**) optical image of internal connections of the MWCNT/CF sensor unit.

The conductive MWCNT/CF composite was fabricated by the dip-coating method, and the procedure is shown in Figure 2. Firstly, the MWCNT solution of 0.6 wt% was prepared by mixing the MWCNT solution (2 wt%) (Chengdu Jiacai Technology Co., Ltd., Chengdu, China) with deionized water. Secondly, the mixture was stirred for 20 min and treated with an ultrasonic cleaner for 10 min to ensure that the MWCNTs were uniformly dispersed in the solution. Thirdly, a piece of CF was rinsed with deionized water to remove impurities and prevent itself from being shrunk after being impregnated by the MWCNT solution. Fourthly, the cleaned and dried CF was immersed in the MWCNT conduction solution of 0.6 wt%, which lasted 10 min, to ensure sufficient adhesion. Finally, the CF, including the MWCNT conductive solution, was dried in an oven at 50 °C for 60 min, and the conductive MWCNT/CF composite was obtained (shown in Figure 3a). Subsequently, the conductive MWCNT/CF was cut into a square with a size of 10 mm × 10 mm.

#### 2.2.2. Encapsulation of the MWCNT/CF Sensor Unit

In our experiment, the diameter and the length of the MWCNT are ~20 nm and 10~30 μm, respectively. The scanning electron microscope (SEM) micrographs (shown in Figure 3b–d) are used to analyze the morphology of the MWCNT/CF composite, which is shown in Figure 3a. It can be seen in the figures that the porous structure of the CF materials is obtained by interspersing threaded yarns between the weft yarns (each consisting of dozens of twisted cotton fibers). Figure 3b,c show that the nanosized MWCNTs are uniformly distributed in the porous structure of the CF and immersed inside the fabric; correspondingly, the three-dimensional conductive network framework of the MWCNT/CF composite could be formed, which would help to gain the MWCNT/CF functional layers with good piezoresistive performance. The high magnification SEM image in Figure 3d demonstrates that the diameter size of the MWCNTs is uniform; most of them are almost 20 nm, and the length of MWCNTs is around 10~30 μm.

The copper–nickel polyester fiber fabric tape has strong electrical conductivity, super adhesion, and flexibility [33]. Two pieces of the copper–nickel polyester fiber fabric tape with dimensions of 9 mm × 9 mm × 0.10 mm were affixed to the upper surface and lower surface of the conductive MWCNT/CF separately, which functioned as the electrode layers. Wires and electrodes should be connected under appropriate temperature and pressure conditions to ensure a stable electrical connection between them. The polyimide (PI) with a thickness of 0.055 mm functioned as the protective layer, and it was used to encapsulate the MWCNT/CF composite and the two electrode layers as an entirety, which is called the MWCNT/CF sensor unit. PI encapsulating ensures the stability and durability of the sensor unit under different environmental conditions [34]. The size of the piezoresistive MWCNT/CF sensor unit is 16 mm × 16 mm × 0.85 mm, as shown in Figure 3e,f. Figure 3g shows an optical image of the internal connection of the sensor unit, where the viscosity of the copper–nickel polyester fiber fabric tape (electrode layers) and the encapsulation of the PI film (protective layers) for the sensing unit ensure a solid electrical connection inside the sensor unit.

### 2.3. Performance Testing

In order to verify the performance of the MWCNT/CF sensor unit fabricated in Section 2.2, the sensitivity, response characteristics, and stability of the sensor unit are tested.

#### 2.3.1. Sensitivity Testing

Sensitivity is very important for the flexible piezoresistive sensor unit to perceive hand motion. The sensitivity of the flexible sensor unit is the ratio of the resistance variation to the corresponding pressure variation, indicating the ability of the sensor to detect external stimuli [35]. The sensitivity (S) shown in Formula (1) [35] can be expressed as follows.

(1) $$S=\delta \left(\frac{\Delta R}{R_{0}}\right)/\delta P$$

where $\Delta R=R_{0}-R$ is the relative variation of resistance of the sensor unit, $R_{0}$ is the initial resistance of the sensor unit without pressure loaded, and δP represents the variation of pressure applied to the sensor unit.

**Figure 4:**
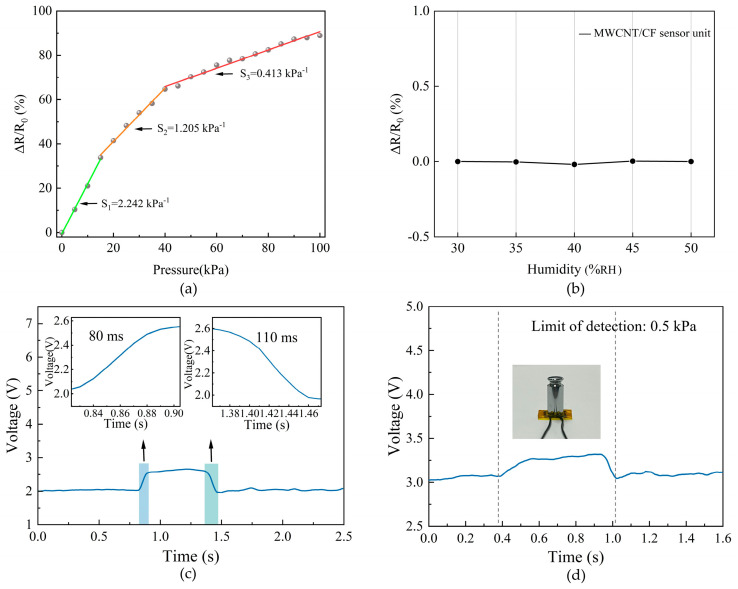
The performance of the MWCNT/CF sensor unit. (**a**) Relationship between the input pressure and the change in relative resistance; (**b**) the resistance response curve at a different humidity; (**c**) response time and recovery time of the sensor unit; (**d**) limit of detection (0.5 kPa).

During sensitivity testing, the pressure of [0 kPa, 100 kPa] with a step of 5 kPa was continuously applied to the MWCNT/CF sensor unit, as shown in Figure 3e. Accordingly, the corresponding resistance variation of the sensor unit was obtained. The relation curve of the pressure and the change in relative resistance are shown in Figure 4a. The sensitivity of the sensor unit has been acquired by means of the segmented linear fitting functions. Figure 4a demonstrates that the sensitivity of the low-pressure range is higher than the high-pressure range. In the pressure range of [0 kPa, 15 kPa], the sensitivity is 2.242 kPa^−1^; in the pressure range of [15 kPa, 40 kPa], the sensitivity is 1.205 kPa^−1^; and in the pressure range of [40 kPa, 100 kPa], the sensitivity is 0.413 kPa^−1^. The piezoresistive layer of the sensor unit is made by the MWCNT/CF with great piezoresistive effect. When there is no pressure on the piezoresistive layer, the sensor unit is in a high impedance state; when the piezoresistive layer is subjected to pressure, it generates obvious compression deformation and, accordingly, obtains high sensitivity; in the high-pressure range, a resistance change in the sensor unit mainly depends on the mutual contact of the conductive fibers inside the MWCNT/CF, which makes the resistance variation decrease; correspondingly, the sensitivity would decrease. The testing results imply that the MWCNT/CF sensor unit has favorable sensitivity within the working range. In this paper, the gestures and motions applied to the sensor unit are all within the pressure range of [0 kPa, 100 kPa], which means that the sensor unit can be applied to gesture recognition.

To detect the effect of humidity on the MWCNT/CF piezoresistive effect, a group of experiments under five different humidity levels were carried out. The relative resistance changes of the MWCNT/CF sensor unit at five different humidity levels are shown in Figure 4b, where just insignificant variations of relative resistance are observed at five different humidity levels. This is mainly due to the great encapsulation effect of the PI film, which makes the MWCNT/CF sensing unit almost unaffected by humidity. The PI film can form a physical barrier that prevents moisture from entering the carbon nanotubes and reduces the interaction of water vapor with the carbon nanotubes. This encapsulation effect limits the influence of humidity on the piezoresistive effect to some extent.

#### 2.3.2. Response Characteristic Testing and Stability Test

In our work, the response characteristic testing includes experiments for both the response time and recovery time of the MWCNT/CF sensor unit. The response time and recovery time indicate the ability to detect stimuli signals and self-recover [35]. To measure the response time and recovery time, a pressure of 25.27 kPa was applied to the sensor unit and released from it. The time-voltage curve is observed, as shown in Figure 4c. When the pressure is loaded on the sensor, the resistance of the sensor unit decreases; meanwhile, the output voltage of the sensor unit gradually increases and reaches the stable value of 2.6 V in about 80 ms, which means that the response time is 80 ms. After the pressure was removed, the resistance of the sensor unit increased; accordingly, the corresponding output voltage would gradually decrease and return to its original voltage of 2 V in about 110 ms, which means that the recovery time is 110 ms.

The performance of the pressure-sensing unit was tested under tiny pressures, as shown in Figure 4d. This demonstrates that the detector detection limit can be as low as 0.5 kPa, which is the pressure generated by a 5 g weight loaded on the pressure sensor. Figure 4d illustrates that the sensor unit has a great ability to detect tiny pressures.

The experimental results show that the MWCNT/CF sensor unit has excellent response characteristics, and it can make a rapid response to external stimuli and promptly return to its initial state. Its response time of 80 ms and recovery time of 110 ms are better than demonstrated in other studies [18,36]. In [18], a composite conductive fabric sensor was proposed with a response time of 700 ms and a recovery time of 1200 ms. In [36], a textile pressure sensor based on carbon black/carbon nanotube polyurethane-coated fabric was designed with a response time of 153 ms and a recovery time of 189 ms.

**Figure 5:**
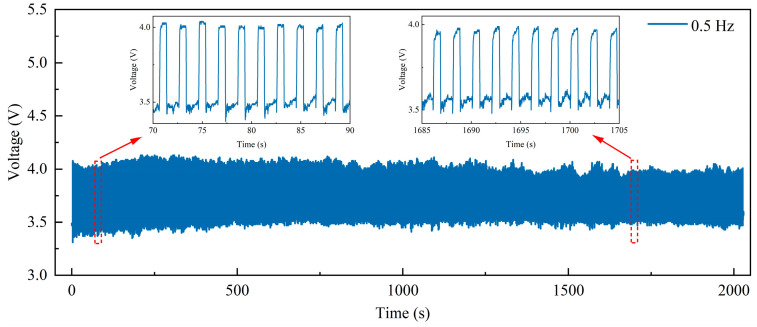
Stability test of the sensor unit and the homologous enlarged images at 70–90 s and 1685–1705 s.

To show the stability of the MWCNT/CF sensor unit, a cyclic pressure loading and unloading test in a pressure range of 0–100 kPa a frequency of 0.5 Hz was performed on the sensor unit. The number of repetitions is approximately 1000 times in about 2000 s, and results are exhibited in Figure 5. In Figure 5, the voltage response curve demonstrates consistent stability, which indicates that the sensor has good stability. Additionally, the homologous enlarged images of 70–90 s and 1685–1705 s show that the voltage response curves at different time periods are almost similar. The study results indicate that conventional loads do not affect the electrical characteristics of the sensor unit, which can ensure stability.

### 2.4. Construction of the Wrist Sensor

#### 2.4.1. Manufacturing of the Wrist Sensor

**Figure 6:**
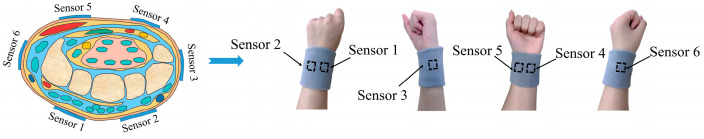
The distribution of the six sensor units on the wristband.

**Figure 7:**
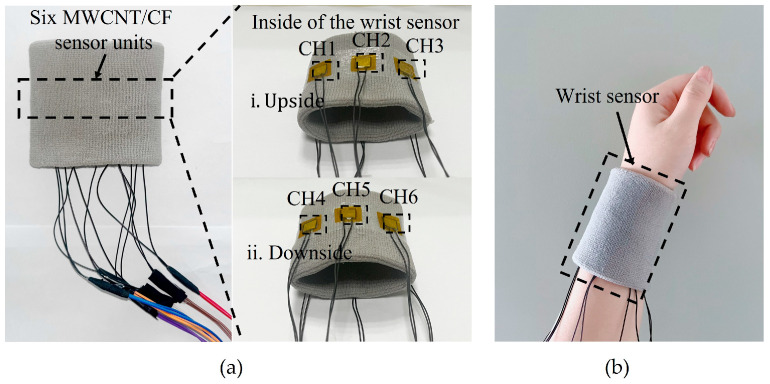
The prototype of the wrist sensor. (**a**) The inside of the wrist sensor with six sensor units; (**b**) an illustration of the wearable wrist sensor.

The above results of performance testing demonstrate that the MWCNT/CF senor unit has great sensitivity, fast response and recovery time, and remarkable stability, and it can be applied to recognize different motions by the output voltage time sequence signals. Based on that, six MWCNT/CF sensor units were fabricated and uniformly fixed on a flexible fabric wristband at specific spots using stretchable medical tape to manufacture a wearable and portable wrist sensor. The original length of the assembled wrist sensor is 8 cm, and it can be stretched up to 12 cm. The repeated experiments have confirmed that the sensor units can securely attach to the wristband and do not negatively affect blood circulation or make the user feel uncomfortable. The results show that with the sensor units mounted on a wristband, the wrist sensor provides sufficient tension to accommodate various wrist sizes and ensure the stability of the sensor and user comfort, and each sensor unit represents one channel. The distribution of the six sensor units on the wristband is shown in Figure 6, and the prototype of the wrist sensor is shown in Figure 7. The six sensor units illustrated in Figure 7 can be easily removed and replaced without damaging the overall structure and have high interchangeability in practical applications. The wrist sensor is very portable and environmentally friendly and can be comfortably worn without interfering with any other movements for users.

When wearing the wrist sensor and performing a gesture or a motion by the wrist or fingers, the corresponding sensor units would be compressed or deform, which would change the resistances of the sensor units from different channels; correspondingly, the output voltages of the sensor units could be detected and obtained, which means that the wrist sensor can recognize different gestures from the detected output signals by connecting to the circuit.

#### 2.4.2. Stability of the Wrist Sensor

**Figure 8:**
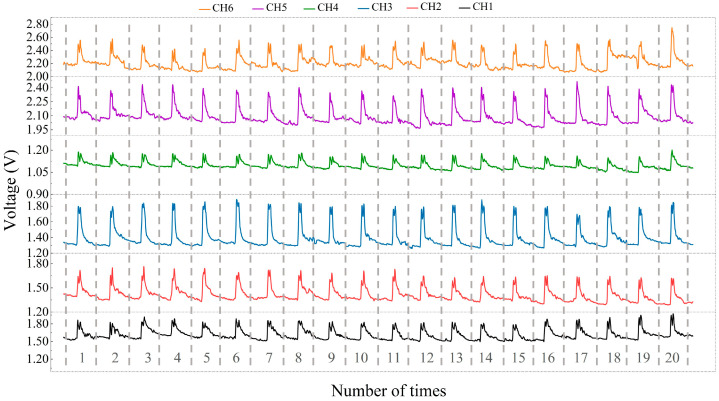
Results of the cyclic tests.

Stability is critical for the wrist sensor to accomplish gesture recognition, and mean value and standard deviation are usually used to express the stability of wrist sensors [37]. A cyclic test was conducted, and the mean values and standard deviations of the six channels (CH1–CH6) from the sensor units were calculated to validate the stability of the wrist sensor. In the experiment, a subject wore the wrist sensor with six channels and performed the international standardized gesture of the letter “E” 20 times while keeping the same intensity of the pressure. The corresponding outputs of the six channels are exhibited in Figure 8. Figure 8 shows that for the gesture performed 20 times, the output of each channel (sensor unit) is uniformly distributed, which means that the stability of the six channels is excellent. Accordingly, the mean values and standard deviations of the output peak values from the six channels are calculated separately and are listed in Table 2. Table 2 implies that the standard deviation is much smaller than the mean value of each channel, which demonstrates that the wrist sensor has great stability and can be applied to detect repetitive behaviors.

## 3. Data Acquisition for Different Gestures

A wrist sensor has been manufactured by integrating six MWCNT/CF sensor units functioning as six channels onto a flexible wristband, which can detect the deformation of muscle and tendon at the human wrist and distinguish different gestures or motions. The gesture signals are normally converted into voltage signals by a microcontroller and transmitted to a computer. Finally, the corresponding output data of voltages for different gestures could be obtained.

**Figure 9:**
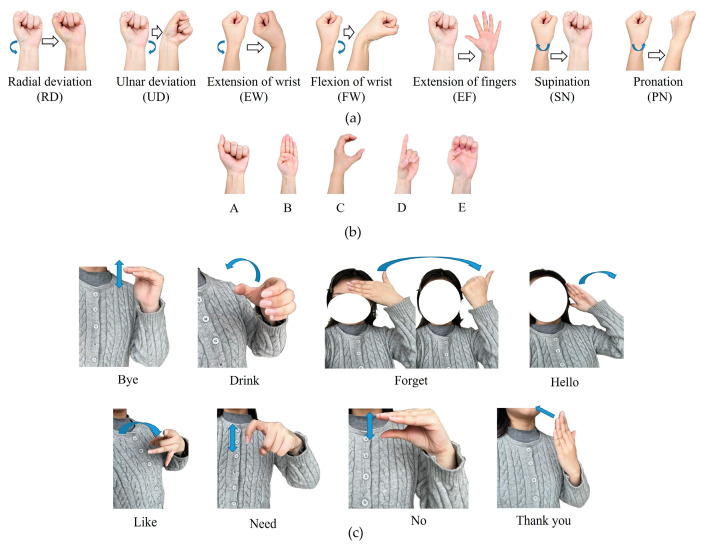
Daily gestures. (**a**) Seven wrist gestures included in Group #1; (**b**) five letter gestures included in Group #2; (**c**) eight daily sign language gestures in Group #3.

Gesture recognition can provide an intuitive, natural, and efficient way of human–computer interaction and is safe and convenient. In the experiment, the subject wore the wrist sensor and performed 20 routine gestures, as shown in Figure 9. Group #1 (shown in Figure 9a) with seven gestures includes radial deviation (RD), ulnar deviation (UD), extension of the wrist (EW), flexion of the wrist (FW), extension of the fingers (EF), supination (SN), and pronation (PN). Group #2 (shown in Figure 9b) includes five international standardized letter gestures: A, B, C, D, and E. In addition, a dataset comprising eight sign language gestures (Group #3) commonly used by deaf individuals daily was collected (as depicted in Figure 9c).

**Figure 10:**
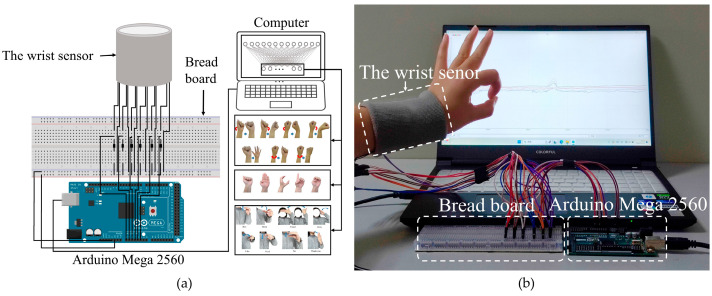
Data acquisition platform. (**a**) A schematic of the gesture signal acquisition device; (**b**) the physical experiment platform.

The subject wore the wrist sensor with six sensor units that are connected to the breadboard by wires, and the breadboard is combined with the Arduino Mega 2560 board (Arduino Co., Ivrea, Italy), which connected to the computer. The platform used to collect the electronic signals is shown in Figure 10. When the subject conducts gestures, the output signals from six channels of the wrist sensor can be acquired by the Arduino Mega 2560 board and recorded in a separate file; meanwhile, gestures can be detected by different voltage signals.

In the experiments, the baud rate of the Arduino board is 115, 200 bit/s and the sampling rate is 100 samples per second (Sa/s). The subject conducted each gesture (shown in Figure 9) 120 times, respectively. There are four subjects, and each subject repeated every gesture 30 times, which means that 120 (4 × 30) samples were collected for each gesture. Correspondingly, the output voltage signals of the three groups’ gestures from the six channels were collected, respectively, which means that a total of 840 gesture samples for Group #1, a total of 600 gesture samples for Group #2, and a total of 960 gesture samples for Group #3 were obtained. After completing the gesture data collection, a total of 2400 gesture samples were collected. Each gesture sample consists of 1200 (200 × 6) voltage features collected from the six channels. Therefore, the dimension of the 840 gesture samples for Group #1 is 840 × 1200, the dimension of the 600 gesture samples for Group #2 is 600 × 1200, and the dimension of the 960 gesture samples for Group #3 is 960 × 1200.

**Figure 11:**
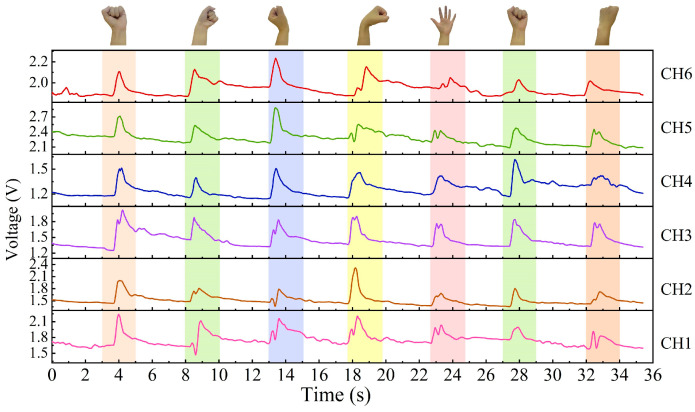
The output signals of the six channels for the seven gestures in Group #1.

**Figure 12:**
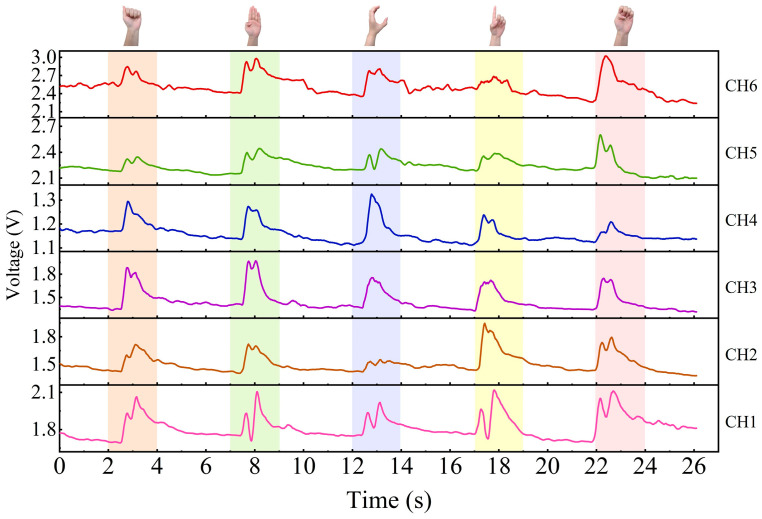
The output signals of the six channels for the five gestures in Group #2.

**Figure 13:**
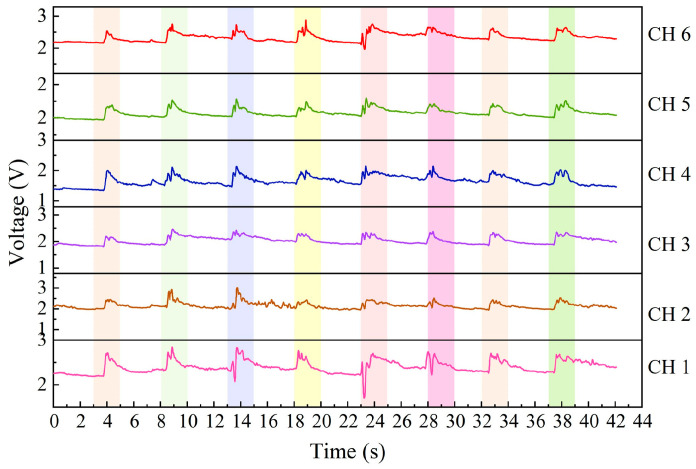
The output signals of the six channels for sign language gestures in Group #3.

When a gesture is performed with the wrist sensor, it usually involves specific movements of tendons and muscles near the sensor unit. Figure 11, Figure 12 and Figure 13 exhibit the output signals of the six channels for the wrist sensor based on the seven, five, and eight gestures from the three groups to illustrate the differences in output signals generated by different gestures. It can be seen in Figure 11, Figure 12 and Figure 13 that each gesture took 2 s, including the process (shown in the colored area) of the original state of the channels to the state of holding the gesture and returning to the original state. This means that the output voltages of the channels for each gesture start to increase from the original state, reach the peak value at the spot that the gesture is held, and finally return to the original state when the gesture is released. This depends on the principle that the corresponding sensor units would be compressed, and the resistances of the sensor units decrease accordingly when the wrist or fingers are flexed and extended, followed by an increase in the output voltages, approaching the peak value; as the gesture is released, the compressed sensor units gradually return to their original state, which leads to a decrease in the output voltages. The voltage responses of the six sensor units (channels) are mainly caused by the contraction or extension of the wrist or fingers for gestures. Figure 11, Figure 12 and Figure 13 demonstrate that output signal curves from the six channels for each gesture are significantly different. These distinctions can be utilized to detect and distinguish different gesture motions.

In our work, data preprocessing is conducted on the collected feature samples. Firstly, the data of 840, 600, and 960 gesture samples are denoised by mean filtering. Then, the z-score normalization method is used to standardize the feature samples by the mean value and the standard deviation. Finally, the processed gesture feature samples are transformed into the distribution with a mean value of 0 and a standard deviation of 1. The z-score computed in Formula (2) [38] is as follows.

(2) $$X^{\prime }=\frac{X_{i}-\mu }{\sigma }$$

where $X_{i}$ is the ith feature value (i = 1, 2, …, 1200), μ is the mean value of $X_{i}$, and σ is the standard deviation of $X_{i}$; in our work, the dimension of each feature sample is 1200.

## 4. Gesture Recognition Based on the CNN-BiLSTM Model

### 4.1. Principle of the CNN-BiLSTM Algorithm

The convolutional neural network (CNN) uses local connectivity and weight sharing to extract internal features in the data at a higher level [39,40]. The CNN in this paper contains convolutional and pooling layers. The convolutional layer is responsible for extracting the features of the input 1200-dimensional time sequence voltage signals, and multiple convolutional kernels are used to perform convolutional operations on the data so as to enhance the features of the original data. The pooling layer is responsible for filtering the outstanding time series features extracted from the convolutional layer, and maximum pooling is used to downscale the time sequence features, which is beneficial in reducing complexity.

**Figure 14:**
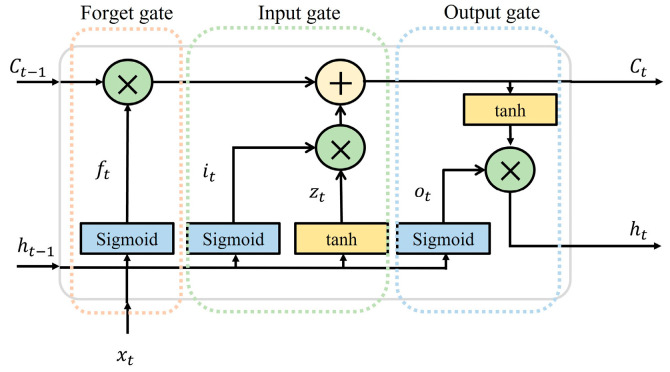
Schematic of the LSTM cell structure.

The advantage of long short-term memory (LSTM) [41] over traditional a recurrent neural network (RNN) is its ability to better handle long sequences and capture long-term dependencies. The LSTM uses gate structures to control the flow of information for better handling of long-term dependencies. Figure 14 illustrates a schematic of one cell for the LSTM memory block. The vital elements of an LSTM network mainly consist of cell state $C_{t}$, forget gate $f_{t}$, input gate $i_{t}$, and output gate $o_{t}$, which are calculated in Formula (3) [42] as follows.

(3) $$lstm\left(\cdot \right)=\left\{\begin{array}{c} i_{t}=sigmoid\left(w_{xi}x_{t}+w_{hi}h_{t-1}+b_{i}\right)\\ f_{t}=sigmoid\left(w_{xf}x_{t}+w_{hf}h_{t-1}+b_{f}\right)\\ z_{t}=tanh(w_{xz}x_{t}+w_{hz}h_{t-1}+b_{z})\\ C_{t}=f_{t}⨀C_{t-1}+i_{t}⨀z_{t}\\ o_{t}=sigmoid(w_{xo}x_{t}+w_{ho}h_{t-1}+b_{o})\\ h_{t}=o_{t}⨀tanh(c_{t}) \end{array}\right.$$

where $x_{t}$ and $h_{t}$ are the input and output vectors at times t and $z_{t}$ is the candidate unit generated by the tanh layer. W ($w_{xi},\;w_{hi},w_{xf},\;w_{hf},\;w_{xz},\;w_{hz},\;w_{xo},\;w_{ho}$) and b ($b_{i},\;b_{f},\;b_{z},\;b_{o}$) are the weight matrices and the bias terms of the corresponding gates.

**Figure 15:**
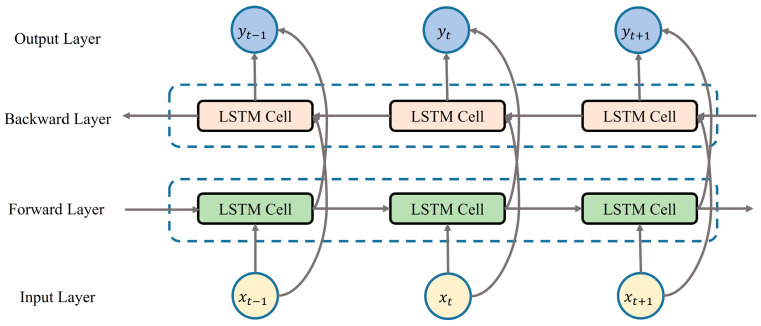
BiLSTM network structure.

Bidirectional long short-term memory [42] (BiLSTM) is used to extract the temporal features from the voltage signal. Compared to traditional LSTM, BiLSTM incorporates an additional reverse LSTM layer to its structure. BiLSTM iteratively processes information in both directions from t=1 to T and from t=T to 1 in order to better capture the temporal correlation between sequential data. This process can consider both past and future information, and its structure is shown in Figure 15.

The computational flow of the BiLSTM is generally described [42] as follows.

In the forward LSTM process of the BiLSTM network structure, the output ${\vec{h}}_{t-1}$ at time t−1 and the current input $x_{t}$ at time t are used as inputs for the forward LSTM layer, and the output ${\vec{h}}_{t}$ [42] of the forward LSTM at time t is obtained.

(4) $${\vec{h}}_{t}={lstm}_{f}(x_{t},{\vec{h}}_{t-1})$$

Similarly, in the reverse LSTM process in the BiLSTM network structure, the output ${\overset{\leftarrow }{h}}_{t+1}$ at time t+1 and the current input $x_{t}$ at time t are utilized as inputs for the backward LSTM layer, and the output ${\overset{\leftarrow }{h}}_{t}$ [42] of the backward LSTM at time t is derived.

(5) $${\overset{\leftarrow }{h}}_{t}={lstm}_{b}(x_{t},{\overset{\leftarrow }{h}}_{t+1})$$

where ${lstm}_{f}(\cdot )$ and ${lstm}_{b}(\cdot )$ are BiLSTM with forward LSTM and reverse LSTM. The output $y_{t}$ [42] of the BiLSTM network at time step t can be formulated as follows.

(6) $$y_{t}=W_{y}\left[{\vec{h}}_{t},{\overset{\leftarrow }{h}}_{t}\right]+b_{y}$$

where $W_{y}$ and $b_{y}$ are the weights and bias matrix of the output layer. The BiLSTM network has a powerful ability to extract temporal features of output signals from the CNN.

The CNN can effectively process time sequence signals to perform feature extraction and dimensionality reduction. LSTM can accomplish temporal feature extraction. The combination of the CNN and BiLSTM networks is used for extracting higher-level temporal features from the time sequence signal obtained from the wrist sensor (Sec. 3), which better accomplishes gesture recognition.

### 4.2. Construction of the CNN-BiLSTM Model

**Figure 16:**
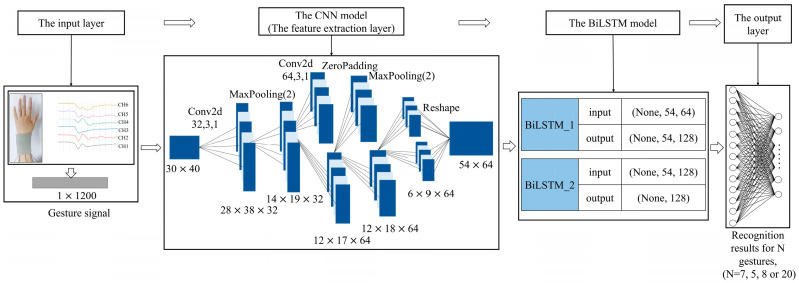
The CNN-BiLSTM network model for the wrist sensor.

A CNN-BiLSTM fusion model is constructed by combining a CNN and a BiLSTM model to recognize different gestures for the wrist senor with six channels based on the preprocessed 840, 600, and 960 gesture samples from the three gesture groups (Group #1, #2, and #3). Each sample includes 1200 feature signals, and it is converted into the shape of 30 × 40 to put into the CNN-BiLSTM model. The CNN-BiLSTM model for the wrist sensor is shown in Figure 16. The input vector for the CNN-BiLSTM model is the 1200 (6 × 200) normalized features of the voltages from the six channels. The input dataset dimensions of the CNN-BiLSTM model based on the samples from Group #1, Group #2, and Group #3 are 840 × 1200, 600 × 1200, and 960 × 1200, respectively. The output of the CNN-BiLSTM model is mapped to the space of sample categories through the fully connected layer to classify and recognize the gestures accurately.

The CNN functioned as the feature extraction layer and consisted of two convolutional layers and two pooling layers. The kernel size and number of kernels for the first convolutional layer are three and thirty-two, respectively, and the values of the second convolutional layer are three and sixty-four. The ReLU is used as the activation function for the convolutional layers, and then the convolutional results are filtered by the pooling layer, which has a pooling kernel size set to 2. Furthermore, it uses a zero-padding layer with the padding parameter set to ((0, 0), (0, 1)) to prevent the information from being lost. Through the CNN network layer, multiple convolution kernels are used to convolve the input preprocessed 1200-dimensional time sequence data. Using the “convolution + pooling” layers, multidimensional feature data are obtained, which are then used as the inputs for the BiLSTM neural network layer. The BiLSTM neural network layer consists of BiLSTM_1 and BiLSTM_2 (both with sixty-four neurons) and is formed by superimposing two BiLSTM neural networks. BiLSTM_2 output is spread to one-dimensional data, and the flattened output data size is 128. To prevent overfitting, a dropout layer with a dropout rate of 0.5 is added after the flattened layer. The final dense layer contains a fully connected layer of N (N = 7, 5, 8, or 20) neurons, with an activation function of Softmax, an input size of 128, and an output size of N.

**Figure 17:**
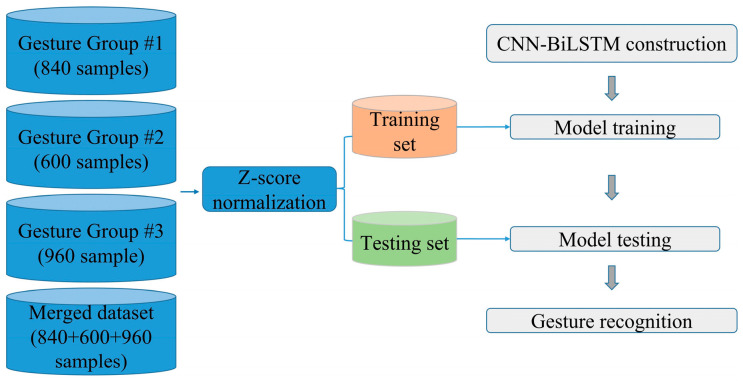
The recognition procedure of gesture recognition based on the CNN-BiLSTM model.

The 840 normalized gesture samples from Group #1, the 600 normalized gesture samples from Group #2, and the 960 normalized gesture samples from Group #3 detected by the wrist sensor are divided into a ratio of 8:2, respectively, to construct the training dataset and testing dataset for the CNN-BiLSTM model. The training dataset samples are used to train the CNN-BiLSTM model. After continuously iterating and fine-tuning the hyperparameters of the model, the testing dataset samples are applied to the trained CNN-BiLSTM model to assess the ability of gesture recognition by the normal evaluation factors. The main procedure of gesture recognition based on the CNN-BiLSTM model for the wrist sensor with six MWCNT/CF sensor units is illustrated in Figure 17.

### 4.3. Evaluation Factors

The evaluation factors are usually used to assess the performance and ability of the network model in prediction tasks. In our work, the accuracy, precision, recall, and F1-score are utilized to evaluate the performance of the CNN-BiLSTM model in recognizing gestures, which is defined in reference [43] as follows.

(7) $$Accuracy=\frac{TP+TN}{TP+FP+FN+TN}$$

(8) $$Precision=\frac{TP}{TP+FP}$$

(9) $$Recall=\frac{TP}{TP+FN}$$

(10) $$F1-score=2\times \frac{Precision\times Recall}{Precision+Recall}$$

where TP represents the samples that are correctly classified as positive samples by the model, TN represents the samples that are correctly classified as negative samples, FP represents the samples that are incorrectly classified as positive samples, and FN represents the samples that are incorrectly classified as negative samples.

### 4.4. Analysis and Discussion of the Recognition Results

During the training process of the CNN-BiLSTM model, the Adam optimizer algorithm is used to update the weight matrix and bias matrix, and the multi-class CrossEntropyLoss function is utilized to optimize the parameters of the model. To increase the iteration speed of the network, the batch size for each sample is set to 32, and the CNN-BiLSTM model has the same parameter values as the CNN layer. The operational flow of the CNN-BiLSTM model and some specific parameters are shown in Figure S1.

To further validate the performance of the CNN-BiLSTM in recognizing different types of gesture signals for the flexible wrist sensor with piezoresistive properties, the LSTM network, and a random forest (RF) model are constructed based on the same dataset as the CNN-BiLSTM model to identify gestures from the three groups. The operational flow of the LSTM and RF models and some specific parameters are shown in Figures S2 and S3.

**Figure 18:**
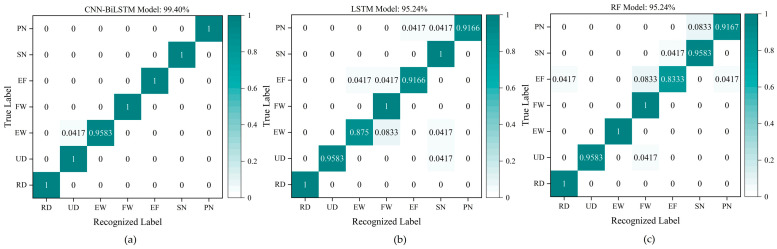
Recognition results for the seven gestures in Group #1. (**a**) The recognition results based on the CNN-BiLSTM model; (**b**) the recognition results based on the LSTM model; (**c**) the recognition results based on the RF model.

For the seven wrist gestures in Group #1, the number of training samples is 672 (840 × 0.8 = 96 × 7), which means there are 96 training samples for each gesture; the number of testing samples is 168 (840 × 0.2 = 24 × 7), which means there are 24 testing samples for each gesture. The features of the 168 testing samples, including seven gestures in Group #1, are extracted and effectively recognized by the trained CNN-BiLSTM model, and the confusion matrix of the recognition results for the seven gestures in 168 testing samples in Group #1 is shown in Figure 18. Figure 18 indicates that the recognition accuracies of the seven gestures (RD, UD, EW, FW, EF, SN, and PN) based on the CNN-BiLSTM are 100%, 100%, 95.83%, 100%, 100%, 100%, and 100%, respectively, and the average recognition accuracy of the seven gestures is 99.40%. The results demonstrate that the CNN-BiLSTM can be applied to distinguish and recognize different wrist gestures by different features based on the signals collected from the wrist sensor fabricated in this paper.

**Figure 19:**
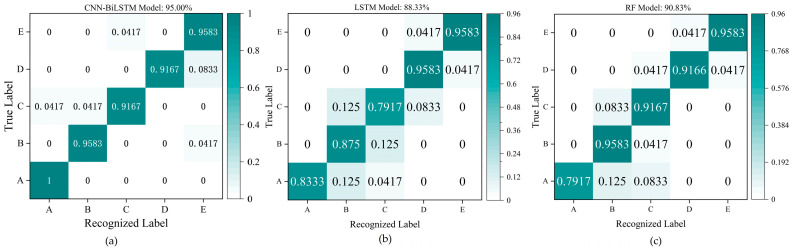
Recognition results for the five gestures in Group #2. (**a**) The recognition results based on the CNN-BiLSTM model; (**b**) the recognition results based on the LSTM model; (**c**) the recognition results based on the RF model.

**Figure 20:**
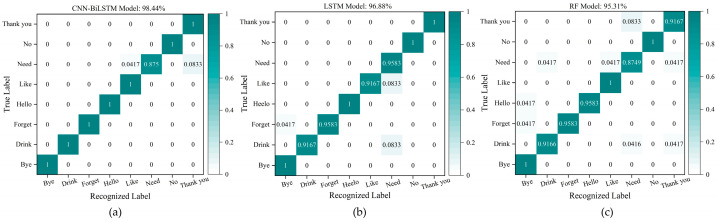
Recognition results for the eight gestures in Group #3. (**a**) The recognition results based on the CNN-BiLSTM model; (**b**) the recognition results based on the LSTM model; (**c**) the recognition results based on the RF model.

For the five letter gestures in Group #2, the number of training samples is 480 (600 × 0.8 = 96 × 5), which means there are 96 training samples for each gesture; the number of testing samples is 120 (600 × 0.2 = 24 × 5), which means there are 24 testing samples for each gesture. The confusion matrix of recognition results for the 120 testing samples based on the same CNN-BiLSTM model used in Figure 18 are shown in Figure 19. Figure 20 exhibits that the recognition accuracies of the five letter gestures (A, B, C, D, and E) are 100%, 95.83%, 91.67%, 91.67%, and 95.83%, respectively, and the average recognition accuracy of the five gestures is 95% The experimental results show that the wrist sensor with six channels can effectively detect the signals for different gestures, and the CNN-BiLSTM model has great performance in feature extraction for different types of gesture signals.

For Group #3, which includes eight daily sign language gestures, the number of training samples is 768 (960 × 0.8 = 96 × 8) and the number of test samples is 192 (960 × 0.2 = 24 × 8). The confusion matrix based on the test set is shown in Figure 20a. The average recognition accuracy of the eight gestures is 98.44%, which means that the research in this paper can realize the accurate recognition of daily sign language gestures. The results of the compared models (LSTM model and RF model) are shown in Figure 20b,c.

Table 3 concludes that the recognition results of accuracy, precision, recall, and F1-score for Group #1 containing seven wrist gestures based on the CNN-BiLSTM are all 99.40%, which are 4.16%, 3.85%, 4.16%, and 4.01% higher than the results based on the LSTM model, and 4.16%, 4.00%, 4.16%, and 4.08% higher than the results based on the RF model. Accordingly, the four evaluation factors in Group #2 containing five letter gestures based on the CNN-BiLSTM model are 95.00%, 95.00%, 95.20%, and 95.10%, respectively, which are 6.67%, 6.06%, 6.87%, and 6.71% higher than the corresponding results based on the LSTM model, and 4.17%, 3.35%, 4.37%, and 4.24% higher than the corresponding results based on the RF model. The four evaluation factors in Group #3 containing eight sign language gestures based on the CNN-BiLSTM model are 98.44%, 98.50%, 98.50%, and 98.50%, which are 1.56%, 1.31%, 1.62%, and 1.47% higher than recognized results based on the LSTM model, and 3.13%, 3.11%, 3.19%, and 3.15% higher than the results based on the RF model. All the results prove that the feature extraction and recognition ability of the CNN-BiLSTM model are superior to the LSTM model and the RF model. This means that the combination of the CNN and the BiLSTM network plays a very important and positive part in feature extraction and recognition for different gesture signals from the wrist sensor.

**Figure 21:**
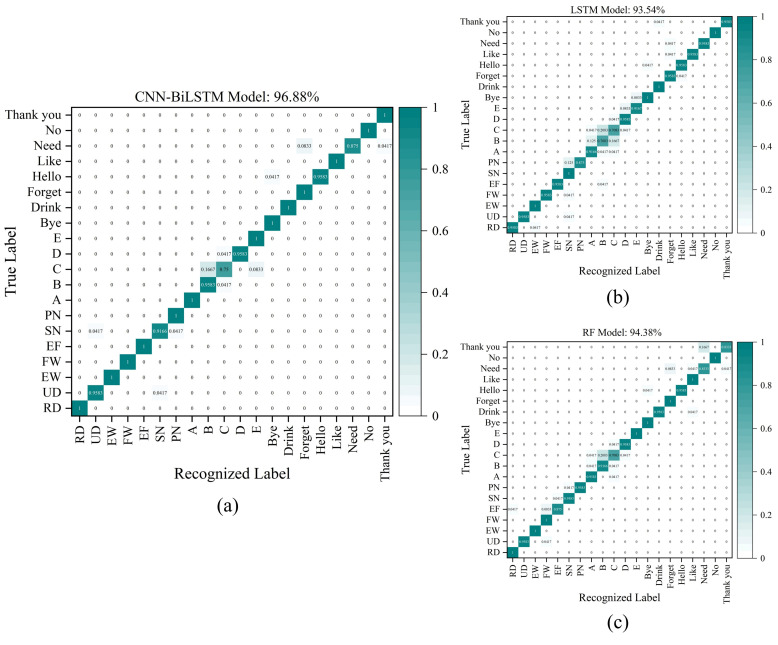
Recognition results for 20 gestures. (**a**) The recognition results based on the CNN-BiLSTM model; (**b**) the recognition results based on the LSTM model; (**c**) the recognition results based on the RF model.

In our work, a total of 2400 samples (7 × 120 + 5 × 120 + 8 × 120 = 2400; there are 120 samples for each gesture) from the 20 gestures, including the extended eight daily sign language gestures, based on the time sequence signal collected by the wrist sensor in Section 3 are used to be recognized using the CNN-BiLSTM model, and the recognition accuracy for the 20 gestures is 96.88% (as shown in Figure 21a). The recognition results of the LSTM model and the RF model based on the same samples as the CNN-BiLSTM model are shown in Figure 21b,c. This shows that the recognition accuracy based on the CNN-BiLSTM model is 3.34% and 2.5% higher than the LSTM model and the RF model. Experimental results show that the wrist sensor proposed in this paper combined with the CNN-BiLSTM model can achieve excellent recognition for different types of gestures.

## 5. Conclusions

A piezoresistive flexible pressure sensor unit is proposed and fabricated based on the flexible MWCNT/CF composite and the dip-coating method, which has an excellent sensitivity of 2.242 kPa^−1^, fast response (80 ms), and significant stability, and it is suitable for sensing and detecting different types of wrist gestures. In the experiments, six MWCNT/CF sensor units were integrated into a flexible wristband to manufacture a portable and wearable wrist sensor. The time-sequential data were collected from the wrist sensor for Gesture Group #1 containing seven wrist gestures, Gesture Group #2 containing five letter gestures, and Gesture Group #3 comprising eight daily sign language gestures, respectively. The CNN-BiLSTM model, the LSTM model, and the RF model have been constructed to classify and recognize different types of gestures in the three groups, respectively. The recognition accuracies of seven wrist gestures in Group #1 based on the three models are 99.40%, 95.24%, and 95.24%, and the recognition accuracies of five letter gestures in Group #2 based on the three models are 95.00%, 88.33%, and 90.83%. Correspondingly, the recognition results of eight gestures in Group #3 based on three models are 98.44%, 96.88%, and 95.31%. Finally, the extended 20 gestures were recognized with an accuracy of 96.88% based on the CNN-BiLSTM model. The results show that the sensor proposed in this paper can effectively detect the feature information of different gestures, and the CNN-BiLSTM model performs very well can effectively capture the features of different signals and realize high-precision recognition of a few gestures or multiple gestures. The CNN-BiLSTM model with its strong ability to extract local and global features from time-sequential signals can effectively leverage spatial features and temporal information and can be widely applied to human gesture recognition based on flexible pressure sensors. All the experiments conducted in this paper provide theoretical support and technical accumulation for human–machine interaction.

## Figures and Tables

**Table 1:** Comparison of the number of gestures, the model used, and recognition accuracy between this work and the previous literature.

Ref.	Materials	Number of Gestures	Recognition Accuracy
This work	MWCNT/CF	20	CNN-BiLSTM (96.88%)
[29]	GNSs/MWCNTs/fabric	5	LSTM (95%)
[30]	Carbon-based e-textile	8	ANN (96.58%)
[31]	Graphene-coated silk–spandex fabric	4	Lenet-5 model (96.07%)
[32]	Graphene aerogel	12	Machine learning (84.7%)

**Table 2:** The mean values and standard deviations of the output peaks from six channels.

Channel	Mean Value (V)	Standard Deviation (V)
CH1	1.8719	0.0367
CH2	1.5908	0.0260
CH3	1.8025	0.0506
CH4	1.2206	0.0117
CH5	2.2579	0.0217
CH6	2.6071	0.0409

**Table 3:** Comparison of the ability of the three models for the gestures from three groups based on the four evaluation factors.

Model	Gesture Group	Accuracy	Precision	Recall	F1-Score
CNN-BiLSTM	Group #1	99.40%	99.40%	99.40%	99.40%
	Group #2	95.00%	95.00%	95.20%	95.10%
	Group #3	98.44%	98.50%	98.50%	98.50%
LSTM	Group #1	95.24%	95.55%	95.24%	95.39%
	Group #2	88.33%	88.94%	88.33%	88.39%
	Group #3	96.88%	97.19%	96.88%	97.03%
RF	Group #1	95.24%	95.40%	95.24%	95.32%
	Group #2	90.83%	91.65%	90.83%	90.86%
	Group #3	95.31%	95.39%	95.31%	95.35%

## Data Availability

The data used to support the study will be available by contacting the corresponding author.

## Supplementary Materials

The following supporting information can be downloaded at: https://www.mdpi.com/article/10.3390/mi15020185/s1, Figure S1: The operational flow of the CNN-BiLSTM model; Figure S2: The operational flow of the LSTM model; Figure S3: The operational flow of the RF model.
