# Supplementary material for: Gesture Recognition Based on a Convolutional Neural Network–Bidirectional Long Short-Term Memory Network for a Wearable Wrist Sensor with Multi-Walled Carbon Nanotube/Cotton Fabric Material
Source: Micromachines (Basel). 2024 Jan 26;15(2):185. doi: 10.3390/mi15020185 (PMC10890478; doi:10.3390/mi15020185)
Supplement: Supplementary file 1 [file micromachines-15-00185-s001.zip › micromachines-2714920-supplementary.pdf]

Supplementary Materials

(1). The operational flow of the CNN-BiLSTM model

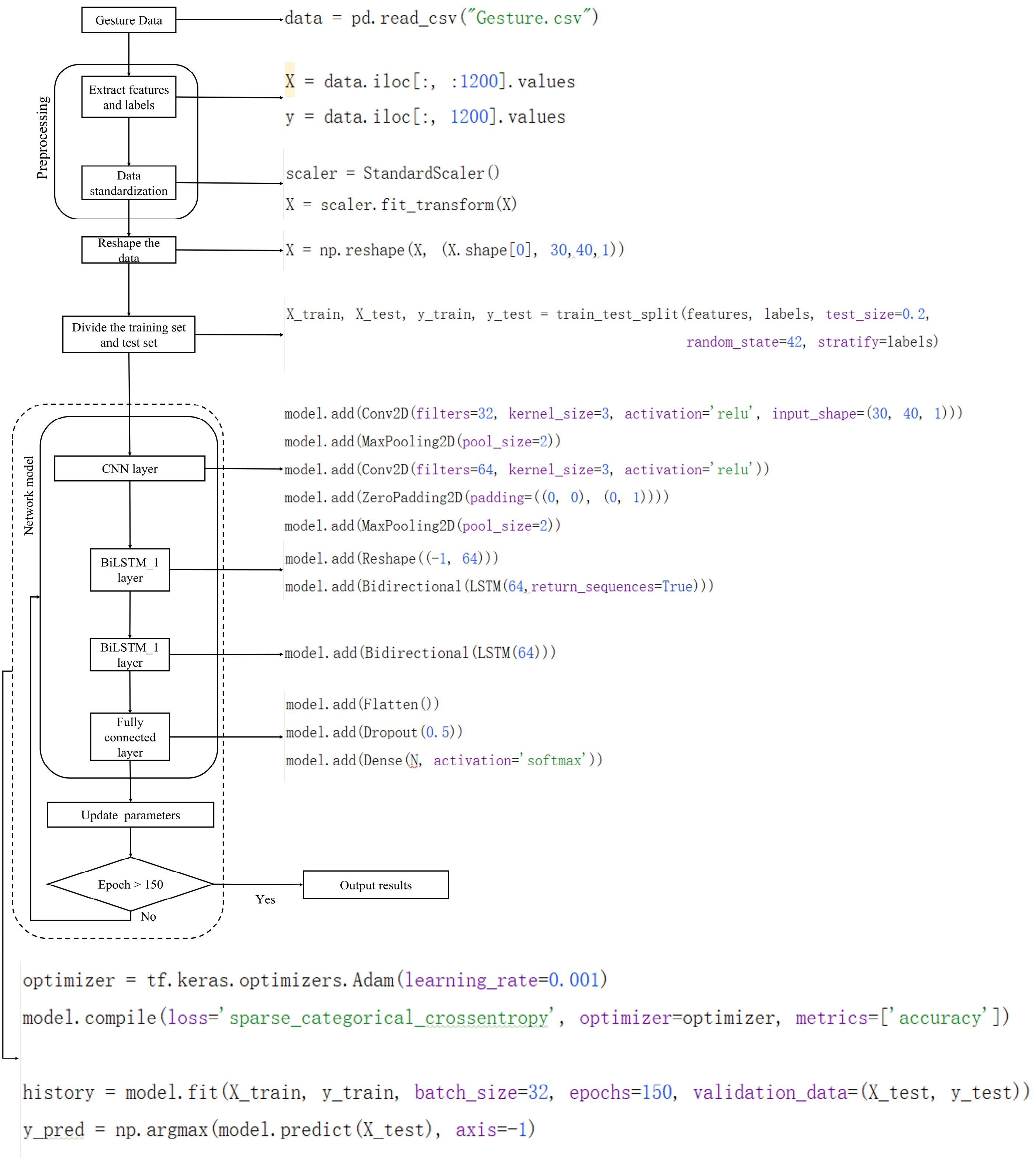

Figure S1. The operational flow of the CNN-BiLSTM model.

Necessary libraries:

```
import pandas as pd
from matplotlib import pyplot as plt
from sklearn.model_selection import train_test_split
```

```
from keras.layers import ZeroPadding2D
from sklearn.metrics import classification_report
from sklearn.preprocessing import StandardScaler
from keras.layers import Dropout
from keras.models import Sequential
from keras.layers import Conv2D, MaxPooling2D, Flatten, Bidirectional, LSTM, Dense, Reshape
from sklearn.metrics import confusion_matrix
import seaborn as sns
```

(2). The operational flow of the LSTM model

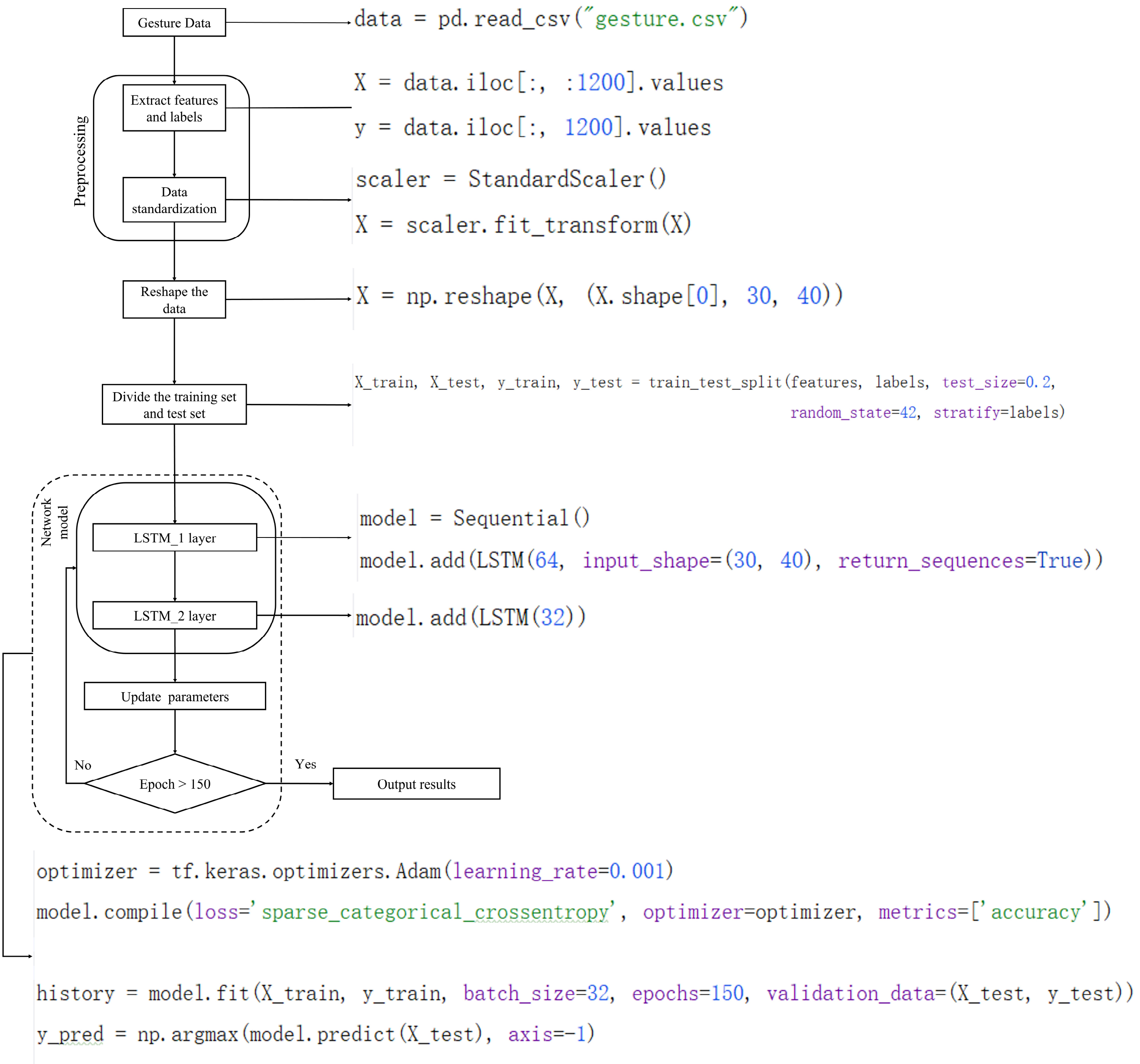

Figure S2. The operational flow of the LSTM model.

(3). The figure of operational flow of the RF model

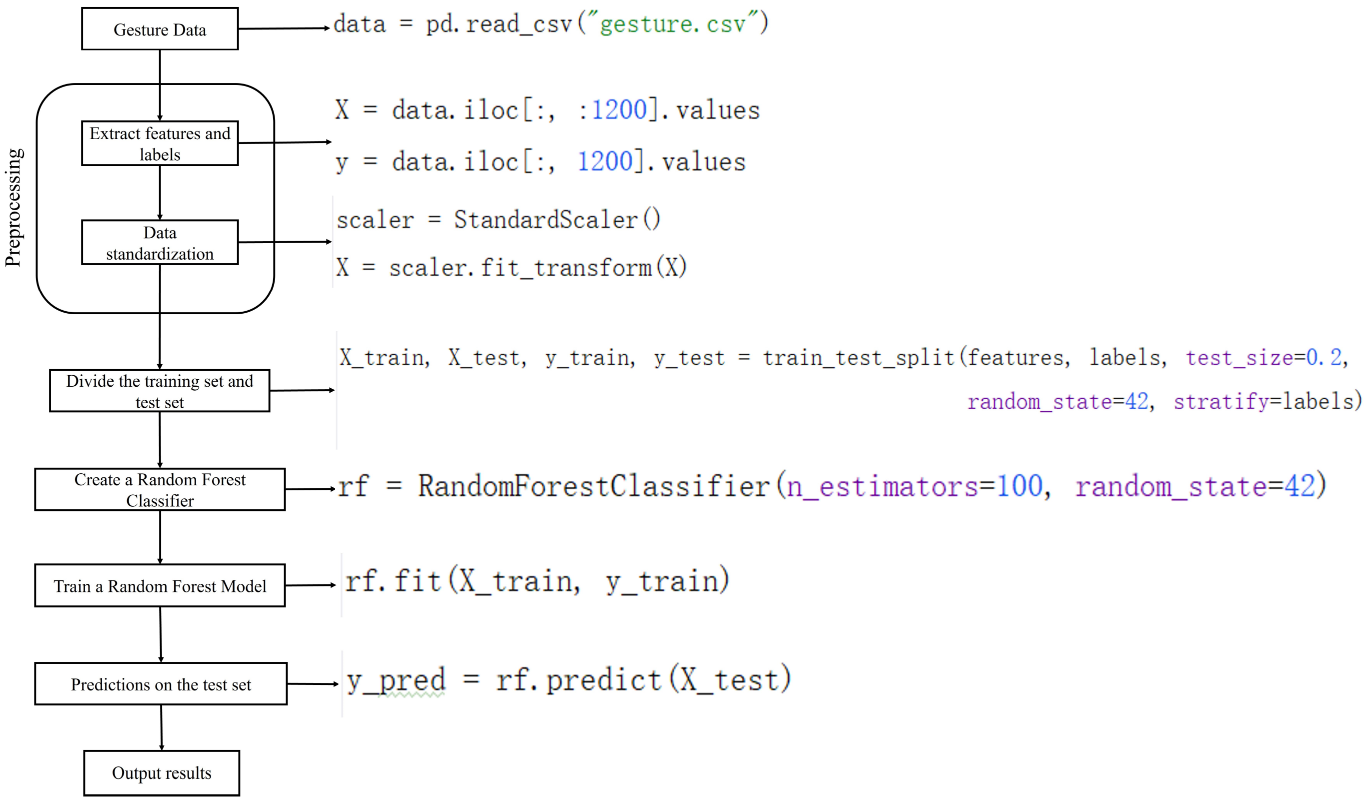

Figure S3. The operational flow of the RF model

Necessary libraries:

import pandas as pd

from sklearn.model_selection import train_test_split

from sklearn.ensemble import RandomForestClassifier

from sklearn.metrics import accuracy_score, precision_score, recall_score, f1_score, classification_report, confusion_matrix

from sklearn.preprocessing import StandardScaler
